# Clinical descriptive measures of shoulder range of motion for a healthy, young and physically active cohort

**DOI:** 10.1186/1758-2555-4-33

**Published:** 2012-09-10

**Authors:** Giampietr L Vairo, Michele L Duffey, Brett D Owens, Kenneth L Cameron

**Affiliations:** 1 Department of Kinesiology, Athletic Training and Sports Medicine Research Laboratory, The Pennsylvania State University, University Park, PA, USA; 2 Department of Kinesiology, Student Fitness Assessment Center, The Pennsylvania State University, University Park, PA, USA; 3 Department of Orthopaedic Surgery, John A Feagin Jr Sports Medicine Fellowship, Keller Army Hospital, United States Military Academy, West Point, NY, USA

**Keywords:** Shoulder, Scapulohumeral, Range of motion, Measurement, Goniometry

## Abstract

**Background:**

The objective of this innovative research study was to describe clinical shoulder complex range of motion (ROM) measures for a young, healthy, and physically active population. This investigation represents a cross-sectional experiment conducted at a military academy-based sports medicine center. Military cadets with no history of shoulder complex injury were assessed within two months of enrollment in the academy; 548 men (18.8 ± 1.0 yr, 75.2 ± 12.2 kg, 178.3 ± 7.4 cm) and 74 women (18.7 ± 0.9 yr, 63.2 ± 8.9 kg, 165.2 ± 6.9 cm) participated. Descriptive measures included cross-body adduction (CAD), flexion (FLX), external rotation (ER0) with the shoulder complex in adduction and elbow flexed to 90°, internal and external rotation (IR, ER) with the shoulder complex at 90° of abduction and elbow flexed to 90° as well as arc (ARC) of IR-ER using standardized clinical quantification techniques. Bilateral and sex differences were evaluated using dependent and independent t-tests, respectively. Percentiles by arm dominance and sex were also calculated for all ROM measures.

**Results:**

Data were normally distributed. Active and passive ROM measures indicated significant bilateral differences (P < 0.05) except for ARC. Sex differences (P < 0.05) were noted for active and passive CAD, FLX and ER0 for the dominant arm as well as active and passive CAD, FLX and ARC for the non-dominant arm.

**Conclusions:**

These original data provide descriptive measures for shoulder complex ROM excursions, assisting sports medicine practitioners in potentially identifying clinical deficiencies and functional outcomes following shoulder injury.

## Background

Clinical range of motion (ROM) assessment is often implemented to objectively evaluate shoulder complex excursion [1]. Various authors have reported shoulder complex ROM measures for broad populations. However, related research studies are associated with limitations that include; smaller sample sizes, inadequately described or selected participant demographics and non-pragmatic experimental methods [2,3]. Thus, existing investigations demonstrate inconsistent results [1], which may be due to prior studies controlling for variables not typically controlled in practical settings [4], thereby diminishing clinical impact and extrapolation to sports medicine practice. Furthermore, although various studies have examined shoulder complex ROM measures in specific athletic populations, most are limited to symptomatic overhead throwing or racquet sport patients, and none have presented descriptive data for a large cohort of young, healthy, and physically active men and women. Moreover, limited evidence exists detailing the influence of arm dominance and sex on associated measures in a related cohort. Therefore, current published shoulder complex ROM descriptive data is of limited utility to sports medicine clinicians for treating athletic patients. Hence, the purpose of this investigation was to describe shoulder complex ROM measures for a young, healthy, and physically active cohort and to examine the effects of arm dominance and sex via a pragmatic study conducted in a clinical setting to replicate the execution of such procedures in sports medicine practice.

## Methods

As part of a larger prospective cohort experimental design [5], a cross-sectional study was conducted to examine shoulder complex ROM in first-year college students entering a military academy. This experiment was approved by the institutional review board prior to its initiation. Within their first two months, 548 men (18.8 ± 1.0 yr, 75.2 ± 12.2 kg, 178.3 ± 7.4 cm) and 74 women (18.7 ± 0.9 yr, 63.2 ± 8.9 kg, 165.2 ± 6.9 cm) with no history of shoulder complex injury underwent bilateral active and passive ROM measures. Based on institutional research data, approximately 70% of all incoming first-year students entering the military academy participated in various varsity athletics in high school. Furthermore, while at the military academy all cadets are required to participate in various intramural, club or intercollegiate athletics each semester and complete fitness assessments twice a year. A detailed description of the physical activity requirements in the study population has been presented elsewhere [6].

We implemented a reliable method [7] for measuring cross-body adduction (CAD). Standard goniometry was used to measure flexion (FLX), external rotation (ER0) with the shoulder complex in adduction and elbow flexed to 90°, internal and external rotation (IR, ER) with the shoulder complex at 90° of abduction and elbow flexed to 90° as well as the arc (ARC) of IR-ER [1,2,4]. Goniometry is a common clinical method used for measuring shoulder complex ROM associated with adequate reliability [1,2,4]. Measurements were conducted by an experienced group of four examiners consisting of sports medicine practitioners with doctoral-level education. Specifically, the examiners represented physical therapists possessing clinical doctorate degrees and sports medicine training. Prior to beginning the study the examiners received standardized training for the employed measurement techniques from one of the group’s senior investigators. Examiners were also blinded to medical history and arm dominance. An individual examiner completed all ROM measures in a standardized manner (Table 1) for each respective participant they assessed. Adopting a standardized method for goniometry has been shown to augment measurement accuracy and precision to acceptable levels [8,9]. Active ROM was measured initially followed by passive. Active ROM defined the maximum excursion attained through voluntary muscle contraction by the participant. Passive ROM defined the maximum excursion attained for the participant until the examiner encountered the respective anatomical limit.

**Table 1:** Standardized shoulder complex ROM measurement techniques

**CAD**	**FLX**	**ER0**	**IR**	**ER**	**ARC**
Participant laying supine with knees flexed. Consisted of measuring the linear distance (cm) between the lateral humeral epicondyle and contralateral acromion process.	Participant laying supine with knees flexed. The axis of the goniometer was placed over the center of the humeral head at the greater tuberosity. The fixed arm was along the participant’s side aligned with the greater trochanter and parallel to the floor using a bubble level. The moving arm was along the lateral aspect of the humeral shaft and aligned with the lateral epicondyle.	Participant laying supine with knees flexed. The humerus was placed in neutral position along the participant’s torso with the elbow in 90° of flexion. The axis of the goniometer was placed over the olecrenon process and aligned with the center of the shaft of the humerus. The fixed arm was positioned perpendicular to the floor using a bubble level. The humerus was then externally rotated and the moving arm was aligned along the shaft of the ulna using the ulnar styloid process as a landmark	Participant laying supine with knees flexed. The humerus was placed in 90° of abduction with the elbow flexed to 90°. The axis of the goniometer was placed over the center of the humeral head at the greater tuberosity. The fixed arm was perpendicular to the floor using a bubble level. The humerus was then internally rotated and the moving arm was aligned along the shaft of the ulna using the ulnar styloid process as a landmark.	Participant laying supine with knees flexed. The humerus was placed in 90° of abduction with the elbow flexed to 90°. The axis of the goniometer was placed over the center of the humeral head at the greater tuberosity. The fixed arm was aligned perpendicular to the floor using a bubble level. The humerus was then externally rotated and the moving arm was aligned along the shaft of the ulna using the ulnar styloid process as a landmark.	The arc of IR and ER was the sum of the IR and ER measurements described above.

Cross body adduction (CAD); flexion (FLX); external rotation (ER0) with the shoulder complex in a neutral position and elbow flexed to 90°; internal and external rotation (IR, ER) with the shoulder complex positioned at 90° of abduction and elbow flexed to 90°; arc (ARC) of IR and ER.

### Statistical analysis

Group means and standard deviations were calculated by arm dominance and sex for all ROM measurements. Probability plots were computed to determine if the data were normally distributed for purposes of satisfying necessary assumptions for t-test analyses. Bilateral and sex differences were evaluated using two-tailed dependent and independent t-tests, respectively. The significance level was set at P<0.05 a priori. Percentiles by arm dominance and sex were also calculated for all ROM measures.

## Results

All data were normally distributed. Active and passive ROM measures indicated significant bilateral differences (P<0.05) with the exception of ARC (Table 2). Sex differences (P<0.05) were noted for active and passive CAD, FLX and ER0 of the dominant arm as well as active and passive CAD, FLX and ARC of the non-dominant arm (Table 3). Data are also presented in percentile fashion for both sexes by arm dominance to indicate quartile cut-points as well as the 5th and 95th percentile for each measure evaluated (Tables 4, 5).

**Table 2:** Shoulder complex ROM comparisons by arm dominance

**Active ROM Excursion**	**Dominant arm**	**Non Dominant arm**	
	**MSD**	**MSD**	**P-value**
CAD (cm)	25.38 ± 3.67	24.70 ± 3.56	<0.001*
FLX (°)	165.56 ± 7.76	164.52 ± 9.33	<0.001*
ER0 (°)	76.46 ± 11.98	74.56 ± 11.65	<0.001*
IR 90° Abduction (°)	54.72 ± 13.35	58.35 ± 11.96	<0.001*
ER 90° Abduction (°)	98.81 ± 10.88	95.30 ± 10.15	<0.001*
ARC (°)	153.53 ± 16.47	153.65 ± 14.58	0.804
**Passive ROM Excursion**	**Dominant arm**	**Non Dominant arm**	
	**MSD**	**MSD**	**P-value**
CAD (cm)	23.31 ± 3.49	22.59 ± 3.42	<0.001*
FLX (°)	170.55 ± 7.68	169.79 ± 9.44	0.009*
ER0 (°)	82.28 ± 12.34	80.48 ± 11.73	<0.001*
IR 90° Abduction (°)	58.91 ± 14.04	62.43 ± 12.80	<0.001*
ER 90° Abduction (°)	104.64 ± 10.83	100.55 ± 9.81	< 0.001*
ARC (°)	163.56 ± 17.41	162.99 ± 15.65	0.251

Cross body adduction (CAD); flexion (FLX); external rotation (ER0) with the shoulder complex in a neutral position and elbow flexed to 90°; internal and external rotation (IR, ER) with the shoulder complex positioned at 90° of abduction and elbow flexed to 90°; arc (ARC) of IR and ER.

Values are mean (M) ± standard deviation (SD); * Indicates statistical significance (P < 0.05).

**Table 3:** Shoulder complex ROM comparisons by sex

**Dominant arm**			
**Active ROM Excursion**	**Men**	**Women**	
	**MSD**	**MSD**	**P-value**
CAD (cm)	25.66 ± 3.59	23.30 ± 3.60	<0.001*
FLX (°)	165.21 ± 7.57	168.12 ± 8.61	0.002*
ER0 (°)	76.03 ± 11.98	79.22 ± 12.13	0.033*
IR 90° Abduction (°)	54.52 ± 13.72	56.03 ± 10.09	0.363
ER 90° Abduction (°)	98.61 ± 10.66	100.14 ± 12.41	0.259
ARC (°)	153.14 ± 16.46	156.16 ± 16.44	0.138
**Non-Dominant arm**			
**Active ROM Excursion**	**Men**	**Women**	
	**MSD**	**MSD**	**P-value**
CAD (cm)	24.94 ± 3.50	22.91 ± 3.55	<0.001*
FLX (°)	164.10 ± 9.46	167.62 ± 7.51	0.002*
ER0 (°)	74.45 ± 11.72	75.35 ± 11.10	0.534
IR 90° Abduction (°)	58.01 ± 12.22	60.91 ± 9.40	0.051
ER 90° Abduction (°)	95.06 ± 10.11	96.99 ± 10.38	0.126
ARC (°)	153.08 ± 14.58	157.89 ± 14.07	0.008*
**Dominant arm**			
**Passive ROM Excursion**	**Men**	**Women**	
	**MSD**	**MSD**	**P-value**
CAD (cm)	23.58 ± 3.40	21.40 ± 3.59	<0.001*
FLX (°)	170.23 ± 7.51	172.85 ± 8.51	0.006*
ER0 (°)	81.83 ± 12.36	85.23 ± 12.32	0.027*
IR 90° Abduction (°)	58.72 ± 14.44	60.20 ± 10.60	0.394
ER 90° Abduction (°)	104.36 ± 10.62	106.55 ± 12.18	0.102
ARC (°)	163.09 ± 17.55	166.76 ± 16.18	0.089
**Non-Dominant arm**			
**Passive ROM Excursion**	**Men**	**Women**	
	**MSD**	**MSD**	**P-value**
CAD (cm)	22.84 ± 3.34	20.72 ± 3.48	<0.001*
FLX (°)	169.43 ± 9.62	172.39 ± 7.52	0.011*
ER0 (°)	80.27 ± 11.85	82.03 ± 10.53	0.227
IR 90° Abduction (°)	62.11 ± 13.06	64.92 ± 10.26	0.076
ER 90° Abduction (°)	100.30 ± 9.73	102.43 ± 10.20	0.079
ARC (°)	162.41 ± 15.66	167.35 ± 14.98	0.011*

Cross body adduction (CAD); flexion (FLX); external rotation (ER0) with the shoulder complex in a neutral position and elbow flexed to 90°; internal and external rotation (IR, ER) with the shoulder complex positioned at 90° of abduction and elbow flexed to 90°; arc (ARC) of IR and ER.

Values are mean (M) ± standard deviation (SD); * Indicates statistical significance (P < 0.05).

**Table 4:** Percentiles for shoulder complex active ROM by arm dominance and sex

**Dominant arm**								
**Active ROM Excursion**	**Sex**	**Percentile**						
		**5**	**10**	**25**	**50**	**75**	**90**	**95**
CAD (cm)	Men	32.00	30.00	28.00	26.00	23.00	21.00	20.00
	Women	28.65	28.00	25.25	23.00	20.00	18.70	16.00
FLX (º)	Men	153.00	155.00	160.00	165.00	170.00	175.00	179.00
	Women	155.00	160.00	164.75	168.00	175.00	180.00	180.00
ER0 (º)	Men	55.00	60.00	70.00	77.00	85.00	90.00	92.00
	Women	55.00	62.80	75.00	80.00	88.50	94.00	95.00
IR 90° Abduction (º)	Men	30.00	38.90	45.00	55.00	65.00	70.00	75.00
	Women	40.00	43.50	49.50	55.00	65.00	69.30	75.00
ER 90° Abduction (º)	Men	80.00	85.00	90.00	100.00	105.00	111.00	115.00
	Women	80.00	84.70	90.00	101.00	110.00	115.00	125.00
ARC (º)	Men	125.00	132.00	143.00	154.00	165.00	175.00	180.00
	Women	128.75	135.00	144.50	155.50	165.25	178.00	181.25
**Non-dominant arm**								
**Active ROM Excursion**	**Sex**	**Percentile**						
		**5**	**10**	**25**	**50**	**75**	**90**	**95**
CAD (cm)	Men	30.00	29.55	27.00	25.00	23.00	20.95	19.00
	Women	28.65	27.50	25.00	22.00	20.00	17.70	16.35
FLX (º)	Men	150.00	155.00	160.00	165.00	170.00	175.00	178.00
	Women	155.00	159.70	164.00	168.00	170.00	180.00	180.00
ER0 (º)	Men	50.00	60.00	67.00	75.00	83.00	90.00	90.00
	Women	60.00	60.00	70.00	76.50	85.00	90.00	90.65
IR 90° Abduction (º)	Men	40.00	44.90	50.00	58.00	65.00	71.10	78.05
	Women	47.00	49.40	50.75	60.00	70.00	71.50	78.65
ER 90° Abduction (º)	Men	78.00	82.00	90.00	95.00	101.00	109.10	111.00
	Women	80.00	85.00	90.00	96.00	105.00	114.30	115.00
ARC (º)	Men	130.00	135.00	144.00	153.00	161.00	171.00	179.55
	Women	130.00	139.00	150.00	155.00	167.00	176.50	182.75

Cross body adduction (CAD); flexion (FLX); external rotation (ER0) with the shoulder complex in a neutral position and elbow flexed to 90°; internal and external rotation (IR, ER) with the shoulder complex positioned at 90° of abduction and elbow flexed to 90°; arc (ARC) of IR and ER.

**Table 5:** Percentiles for shoulder complex passive ROM by arm dominance and sex

**Dominant arm**								
**Passive ROM Excursion**	**Sex**	**Percentile**						
		**5**	**10**	**25**	**50**	**75**	**90**	**95**
CAD (cm)	Men	29.00	28.00	26.00	24.00	21.50	19.00	18.00
	Women	27.00	26.00	24.00	21.00	18.88	17.00	15.00
FLX (º)	Men	159.00	160.00	165.00	170.00	175.00	180.00	185.00
	Women	158.05	163.00	169.50	172.50	180.00	183.60	185.65
ER0 (º)	Men	60.00	65.00	75.00	84.00	90.00	95.00	100.00
	Women	60.00	70.00	80.00	88.00	91.25	100.30	105.00
IR 90° Abduction (º)	Men	35.00	42.00	50.00	60.00	70.00	76.10	80.00
	Women	40.70	45.70	52.00	60.00	68.25	75.00	80.00
ER 90° Abduction (º)	Men	88.95	90.90	96.00	105.00	110.00	117.00	120.05
	Women	86.05	90.00	100.00	108.50	115.00	123.30	130.65
ARC (º)	Men	135.00	140.00	151.00	163.00	175.00	185.00	190.00
	Women	140.00	145.00	155.00	167.50	175.00	187.50	195.50
**Non-dominant arm**								
**Passive ROM Excursion**	**Sex**	**Percentile**						
		**5**	**10**	**25**	**50**	**75**	**90**	**95**
CAD (cm)	Men	28.00	27.00	25.00	23.00	20.00	19.00	17.48
	Women	26.30	24.65	23.00	20.00	18.00	16.00	15.00
FLX (º)	Men	155.00	160.00	165.00	170.00	175.00	180.00	185.00
	Women	160.00	163.70	167.00	173.00	177.25	180.00	185.00
ER0 (º)	Men	56.90	65.00	73.00	80.00	90.00	95.00	95.05
	Women	65.00	68.50	75.00	85.00	90.00	95.00	98.65
IR 90° Abduction (º)	Men	41.00	46.00	53.00	61.50	70.00	77.10	83.05
	Women	50.00	50.70	55.00	65.00	72.75	80.00	84.65
ER 90° Abduction (º)	Men	85.00	88.90	95.00	100.00	107.00	115.00	115.05
	Women	86.75	90.00	95.00	103.00	110.25	120.00	120.00
ARC (º)	Men	138.00	143.00	152.00	162.00	172.00	181.00	190.00
	Women	143.00	145.00	158.75	167.00	175.25	189.00	198.50

Cross body adduction (CAD); flexion (FLX); external rotation (ER0) with the shoulder complex in a neutral position and elbow flexed to 90°; internal and external rotation (IR, ER) with the shoulder complex positioned at 90° of abduction and elbow flexed to 90°; arc (ARC) of IR and ER.

## Discussion

Our results demonstrate greater FLX and ER regardless of abduction angle as well as less IR in the dominant compared to non-dominant arm. These findings contrast those of Barnes et al. [1] that reported no significant bilateral FLX differences in a broad participant population, yet parallel the findings of Johnson [10] for a college-aged athletic cohort. Factors that may underpin increased FLX for the dominant arm in a related cohort are not currently completely understood, but this phenomenon has been suggested to potentially arise from functional adaptations that permit greater ROM available for the purpose of executing various sports-related tasks, such as overhead throwing [10]. However, similar to Barnes et al. [1], our results yielded greater active and passive ER regardless of abduction angle as well as less IR for the dominant compared to non-dominant arm. A trend for greater ER and lesser IR in the dominant arm has been previously described for a college-aged athletic population [3,11,12] and may be attributed to functional adaptions associated with participation in sports [13], especially overhead related activities [14]. Furthermore, our results are in agreement with Gunal et al. [2] who noted greater active and passive CAD in the non-dominant compared to dominant arm. Less CAD in the dominant arm is yet another previously observed trend [15] in a young athletic population and has been linked to posteroinferior glenohumeral joint capsular stiffness [16,17], which potentially represents an adaptive phenomenon typically associated with athletic throwing activities [16]. Total active, as well as passive, ARC of IR and ER measured in our cohort were comparable bilaterally, thereby complementing previous findings [7,18,19]. Shoulder pain has been identified as a significant factor decreasing total ARC of IR and ER for the dominant compared to non-dominant arm in athletes [19]. None of our participants reported pain, which may explain why no bilateral differences were found.

Contrary to a prior report [20], our data suggest sex may also influence shoulder complex ROM in an athletic cohort. Greater active and passive FLX and ER0 for the dominant arm as well as greater active and passive FLX and ARC for the non-dominant arm were observed among women compared to men in our investigation, which is comparable to Barnes et al. [1]. Women also exhibited greater bilateral measures of active and passive CAD than men in our study. The influence of sex on shoulder complex ROM measures has been described before [1,21] and may be associated with muscle morphology differences between men and women [21]. Roy et al. [21] demonstrated an inverse relation between shoulder complex muscular strength and ROM, and suggested that increased muscle bulk, indicative of greater strength in asymptomatic men compared to women, may yield decreased ROM. Barlow et al. [22] reported similar observations among healthy, young bodybuilders and non-bodybuilders, and proposed that the observed results were attributed to group differences in muscle mass.

Although we identified similarities and differences for our study and previous reports, comparing our results with prior findings is difficult due to considerable differences in participant populations and methods. Notable sources of variability between our study and preceding investigations are mostly attributed to discrepancies in experimental design and participant demographics. Nonetheless, our study is novel in presenting clinically relevant descriptive data for healthy, young and physically active adults, which represents the population typically treated by sports medicine practitioners. Thus, while our findings are limited in generalizability to other populations, they are well suited for descriptions of young athletes.

Determining descriptive values for an athletic population is essential to providing sports medicine practitioners with a frame of reference with which to interpret clinical measures obtained from respective patients. Such data are also necessary for evaluating patient progression through treatments to determine clinical intervention efficacy. Our investigation indicates that traditional shoulder complex ROM examinations and clinical interpretations, which do not account for arm dominance and sex factors, may be ambiguous in an athletic cohort. These findings suggest shoulder complex ROM differences, both bilaterally as well as among men and women, may be at least partially explained by arm dominance and sex in young, healthy and physically active adults. Therefore, influences of arm dominance and sex should be considered when shoulder complex ROM is clinically evaluated in a similar population.

We observed statistically significant differences for several of the ROM measures examined in our study by arm dominance and sex, yet whether or not these differences are clinically important remains unclear. Due to the large number of observations and associated statistical power, it is likely that even small mean differences reached statistical significance in our study. In fact, the largest mean differences were no greater than 2.5cm for CAD or 5° for any of the goniometric measurements. It has been reported previously that mean differences less than 7° [23] or 8° [24] for goniometric measurements may lack clinical relevance, but limited empirical evidence is available to support these values as minimum clinically important differences. The American Medical Association [25] suggests that differences less than 10° at the shoulder complex may be regarded as clinically insignificant when evaluating permanent impairment. However, much smaller differences may be clinically significant in high level athletes, particularly in sports with considerably upper extremity demands. Thus, further study is needed to empirically quantify minimum clinically important differences for shoulder complex ROM in young athletes.

Our study was associated with certain limitations. One-dimensional goniometric measurements do not concomitantly assess multi-planar joint excursions, which may contribute to the respective resultant net ROM measurement. Thus, more sophisticated, yet non-pragmatic, instrumentation, such as photogrammetry, may be better equipped to comprehensively capture ROM measures. We were also unable to estimate intra- and inter-rater reliability within and between examiners who assessed ROM using standardized procedures, which is an important limitation when interpreting the results of our study. However, we relied on prior reliability estimates [4,7,9,26,27] for the shoulder complex ROM measures studied. Furthermore, all clinicians completed standardized training prior to data collection to enhance consistency between and within examiners [8,9]. Although these suggested drawbacks represent important limitations to the internal validity of our study, our measures represent those used in real world settings to assess patients, which considerably contribute to the external validity and generalizability of these results to clinical sports medicine [4]. Continued investigation is necessary to confirm these findings and further contribute to the literature on shoulder ROM in young athletic populations.

## Abbreviations

ROM: Range of motion; CAD: Cross-body adduction; FLX: Flexion; ER0: External rotation with the shoulder complex in adduction and elbow flexed to 90°; IR: Internal rotation with the shoulder complex at 90° of abduction and elbow flexed to 90°; ER: External rotation with the shoulder complex at 90° of abduction and elbow flexed to 90°; ARC: Arc of internal and external rotation.

## Competing interests

We declare to have no competing interests.

## Authors’ contributions

GLV contributed to the interpretation of data analysis, dissemination of findings as well as concept and construct of the related manuscript. MLD, BDO and KLC equally contributed to the experimental design, collection and analysis of data, dissemination of findings as well as review of the related manuscript. All authors read and approved the final manuscript.
